# Efficacy and safety of CHF6001, a novel inhaled PDE4 inhibitor in COPD: the PIONEER study

**DOI:** 10.1186/s12931-020-01512-y

**Published:** 2020-09-22

**Authors:** Dave Singh, Aida Emirova, Catherine Francisco, Debora Santoro, Mirco Govoni, Marie Anna Nandeuil

**Affiliations:** 1 Medicines Evaluation Unit, The University of Manchester, Manchester University NHS Foundation Trust, Manchester, UK; 2 Global Clinical Development, Chiesi, Parma, Italy

**Keywords:** Acute exacerbations of COPD, Chronic obstructive pulmonary disease, Chronic bronchitis, Phosphodiesterase inhibitors

## Abstract

**Background:**

This study evaluated the efficacy, safety and tolerability of the novel inhaled phosphodiesterase-4 inhibitor CHF6001 added-on to formoterol in patients with chronic obstructive pulmonary disease (COPD).

**Methods:**

Randomised, double-blind, placebo- and active-controlled, parallel-group study. Eligible patients had symptomatic COPD, post-bronchodilator forced expiratory volume in 1 s (FEV_1_) 30–70% predicted, and history of ≥1 moderate/severe exacerbation. Patients were randomised to extrafine CHF6001 400, 800, 1200 or 1600 μg twice daily (BID), budesonide, or placebo for 24 weeks. Primary objectives: To investigate CHF6001 dose-response for pre-dose FEV_1_ after 12 weeks, and to identify the optimal dose. Moderate-to-severe exacerbations were a secondary endpoint.

**Results:**

Of 1130 patients randomised, 91.9% completed. Changes from baseline in pre-dose FEV_1_ at Week 12 were small in all groups (including budesonide), with no CHF6001 dose-response, and no significant treatment–placebo differences. For moderate-to-severe exacerbations, CHF6001 rate reductions versus placebo were 13–28% (non-significant). In *post-hoc* analyses, CHF6001 effects were larger in patients with a chronic bronchitis phenotype (rate reductions versus placebo 24–37%; non-significant), and were further increased in patients with chronic bronchitis and eosinophil count ≥150 cells/μL (49–73%, statistically significant for CHF6001 800 and 1600 μg BID). CHF6001 was well tolerated with no safety signal (including in terms of gastrointestinal adverse events).

**Conclusions:**

CHF6001 had no effect in the primary lung function analysis, although was well-tolerated with no gastrointestinal adverse event signal. *Post-hoc* analyses focused on exacerbation risk indicate specific patient subgroups who may receive particular benefit from CHF6001.

**Trial registration:**

ClinicalTrials.gov (NCT02986321). Registered 8 Dec 2016.

## Background

Phosphodiesterase-4 (PDE4) inhibition has an established role in the management of chronic obstructive pulmonary disease (COPD); roflumilast is an anti-inflammatory drug that prevents exacerbations in the subgroup of patients with a chronic bronchitis phenotype [1]. Roflumilast is taken orally and consequently is associated with a higher incidence of systemic adverse events related to PDE4 inhibition including diarrhoea, nausea, weight loss and abdominal pain, resulting in both substantial treatment discontinuation in clinical practice and withdrawal from clinical trials [1–7].

CHF6001 is a novel inhaled PDE4 inhibitor [8, 9] that has been developed as an extrafine formulation (i.e., with mass median aerodynamic diameter ≤ 2 μm) and to have low systemic exposure. This allows CHF6001 to reach a therapeutic concentration in the target organ, the lung, with reduced systemic exposure, limiting systemic adverse effects. Indeed, CHF6001 inhaled twice daily (BID) has previously demonstrated lung-targeted anti-inflammatory effects in patients with COPD and a chronic bronchitis phenotype [10, 11].

The aim of this study was to evaluate the efficacy, safety and tolerability of CHF6001 in patients with COPD when added on to a bronchodilator (formoterol fumarate), and to identify the optimal dose(s) of CHF6001 for further development. In addition to reporting the pre-specified results of the study, this manuscript presents the results from a series of *post-hoc* analyses that explored the effect of CHF6001 (in terms of moderate-to-severe exacerbations) in various patient subgroups.

## Methods

### Trial design and participants

This was a multicentre, randomised, double-blind, double-dummy, placebo- and active-controlled, parallel-group, dose-ranging study. Eligible patients were at least 40 years of age, with a diagnosis of COPD, a smoking history of at least 10 pack-years (current and ex-smokers were eligible), post-bronchodilator forced expiratory volume in 1 s (FEV_1_) 30–70% predicted, a history of at least one moderate or severe exacerbation in the previous 12 months, symptomatic (modified Medical Research Council dyspnoea score ≥ 2 and COPD Assessment Test score ≥ 10), and receiving daily maintenance therapy with an inhaled corticosteroid (ICS) and a long-acting β_2_-agonist (LABA) at a stable dose and regimen for at least 2 months prior to entry. Key exclusion criteria were: a diagnosis of asthma or other respiratory disease that might impact data interpretation; and a moderate or severe exacerbation in the 8 weeks prior to study entry. All patients provided written informed consent prior to any study-related procedure. Full inclusion and exclusion criteria are listed in Additional file 1.

Patients who met the inclusion and exclusion criteria entered a two-week run-in period during which they received formoterol fumarate 12 μg BID plus salbutamol as required. At the baseline visit, patients were randomised equally to one of six treatment groups: one of four extrafine CHF6001 doses (400, 800, 1200 or 1600 μg BID) via dry powder inhaler (DPI), budesonide 400 μg BID via a different DPI, or placebo. All patients continued to receive formoterol fumarate BID and salbutamol as required throughout the study. Patients were assigned to treatment centrally via interactive voice response technology, using a balanced block randomisation scheme stratified by site. Patients, investigators, and site and sponsor staff were blinded to treatment assignment by use of a double-dummy design, with matching placebo to CHF6001 DPI, and matching placebo to budesonide DPI.

Patients returned to the study site for visits after 3, 6, 12, 18 and 24 weeks, when data were captured from pre-dose spirometry (slow vital capacity manoeuvres for inspiratory capacity [IC] and forced vital capacity [FVC] manoeuvres for FEV_1_ and FVC), and from the Transition Dyspnea Index (TDI) and St George’s Respiratory Questionnaire (SGRQ). Patients completed an electronic diary daily, in which they recorded symptoms (using the Exacerbations of Chronic Pulmonary Disease Tool – Respiratory Symptoms [E-RS]) and rescue medication use. The occurrence of exacerbations was captured throughout the study, with moderate exacerbations defined as those requiring treatment with systemic corticosteroids and/or antibiotics, and severe exacerbations requiring hospitalisation or resulting in death. Adverse events (AEs) were captured throughout the study, with safety evaluated using haematology, blood chemistry, urinalysis, vital signs and 12-lead electrocardiograms (ECG).

The study was approved by the independent ethics committees or research boards at each institution, and was performed in accordance with the principles of the Declaration of Helsinki, and the International Conference on Harmonisation notes for guidance on Good Clinical Practice (ICH/CPMP/135/95). There were no substantial protocol amendments that impacted recruited patients. Study registration: ClinicalTrials.gov (NCT02986321).

### Outcomes

The primary objectives of the study were to investigate the dose-response relationship of CHF6001 with respect to pre-dose FEV_1_ after 12 weeks, and to identify the optimal dose of CHF6001. Secondary objectives were to compare CHF6001 with placebo and with budesonide over 24 weeks in terms of: pre-dose FEV_1_, FVC and IC, TDI focal score, SGRQ total score, E-RS total score, rescue medication use, and the rate of moderate-to-severe exacerbations. Safety and tolerability were also monitored as a secondary objective.

As exploratory objectives, the effects of CHF6001 versus placebo were evaluated on systemic C-reactive protein (CRP), fibrinogen, surfactant protein D (SP-D), club cell protein 16 (CC-16), interleukin-6 (IL-6), IL-8 and blood eosinophil count, using the same methods as presented in Singh et al [10].

### Sample size and statistical methods

The study was powered on the slope of line obtained by regressing pre-dose FEV_1_ change at Week 12 (the primary endpoint) against dose (i.e., linear dose-response) [12]. It was estimated that 735 evaluable patients (147 in each of the CHF6001 and placebo groups) would be sufficient to reject the null hypothesis that the slope equalled zero with an 80% power and a two-sided alpha level of significance of 0.05, assuming a standard deviation of 240 mL and that a change of 70 mL would be reached with CHF6001 1600 μg BID. The same number of evaluable patients was included in the budesonide group. The power of the calculated sample size to fit a significant E_max_ model was estimated across 1 thousand simulations to be 87.5%. Given an estimated drop-out rate of 20%, it was planned to randomise 1102 patients to obtain 882 evaluable patients.

The dose-response relationship of CHF6001 was explored using linear, E_max_, and linear-log models. Change from baseline in pre-dose morning FEV_1_ was analysed using a linear mixed model for repeated measures (MMRM) including treatment, visit, treatment by visit interaction and sites pooled by country, as effects, and baseline FEV_1_ value and baseline by visit interaction as covariates. Most of the secondary efficacy endpoints were analysed using a similar MMRM to that used for the primary efficacy endpoint. Biomarker data (log-transformed values) were analysed using an analysis of covariance model including treatment and sites pooled by country as fixed effects, and the baseline value as covariate. The moderate-to-severe COPD exacerbation rate was analysed using a negative binomial model including treatment and sites pooled by country as factors, and logarithm of time into the study as an offset; the adjusted exacerbation rates in each group and the adjusted rate ratios versus placebo were estimated by the model. In addition, a series of hypothesis-generating *post-hoc* analyses were performed on the moderate-to-severe COPD exacerbation rate data using the same model in three subgroups: patients with a chronic bronchitis phenotype; using a blood eosinophil value threshold at baseline of 150 cells/μL; and patients with a combined chronic bronchitis phenotype and blood eosinophil level ≥ 150 cells/μL at baseline. All data were analysed using Statistical Analysis System software Version 9.4.

The intention-to-treat (ITT) population, defined as all randomised patients who received at least one dose of study medication and who had at least one available post-baseline efficacy evaluation, was used for all efficacy evaluations. Safety evaluations were performed on the safety set, which was all randomised patients who received at least one dose of study medication.

## Results

### Participants

**Fig. 1:**
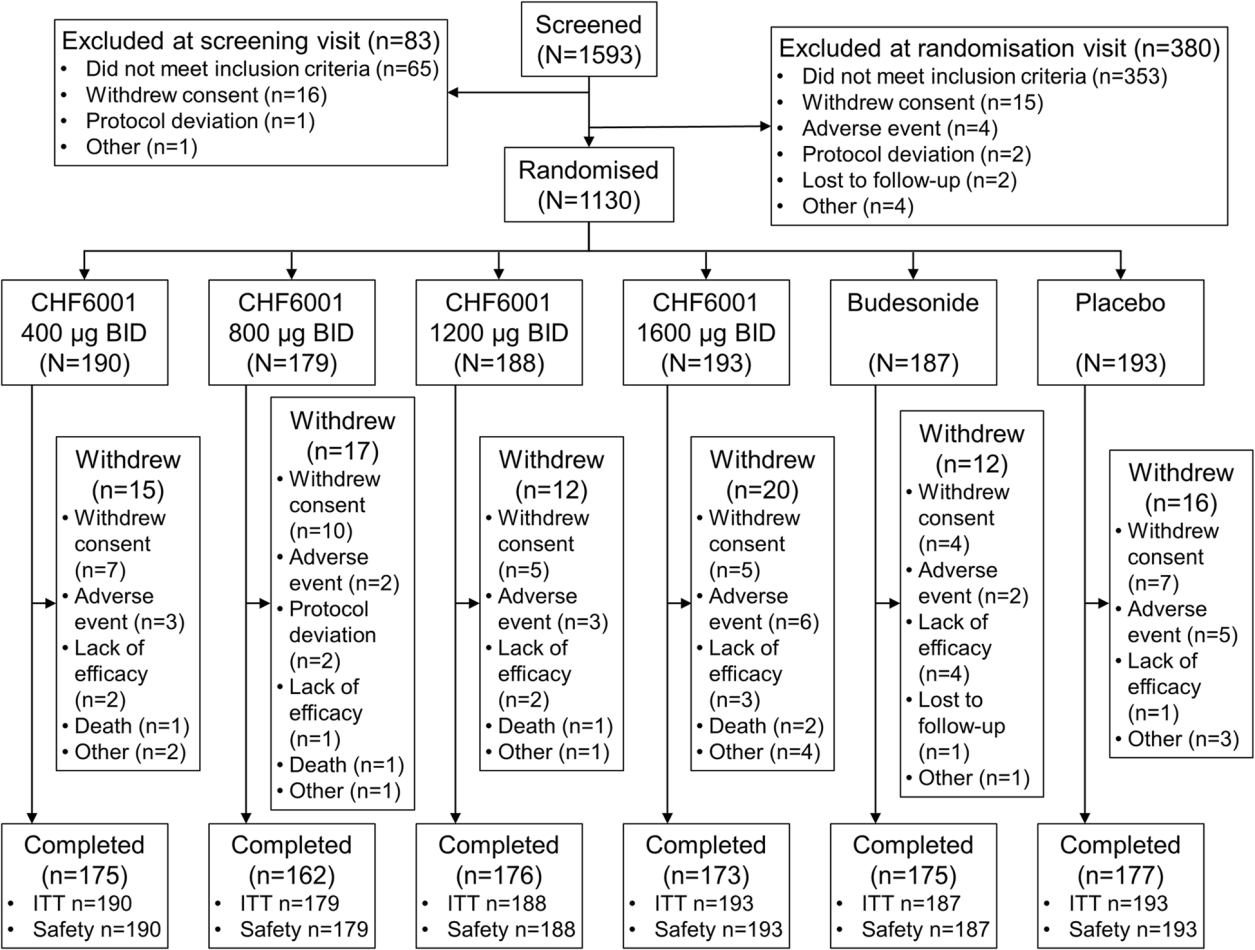
Patient disposition. BID, twice daily; ITT, intention-to-treat

The study was conducted between 15 December 2016 and 9 January 2018 in seven countries (Bulgaria, Germany, Hungary, Poland, Russia, Ukraine and United Kingdom). Of 1130 patients randomised, 1038 (91.9%) completed the study, with the proportion of patients withdrawing (and the reasons for withdrawal) similar in each group (Fig. 1). Baseline demographics and disease characteristics were similar between groups (Table 1).

**Table 1:** Baseline demographics and disease characteristics

	CHF6001				Budesonide 800 μg (***N*** = 187)	Placebo (***N*** = 193)	Overall (***N*** = 1130)
	400 μg BID (***N*** = 190)	800 μg BID (***N*** = 179)	1200 μgBID (***N*** = 188)	1600 μg BID (***N*** = 193)			
Age (years)	64.0 (8.5)	65.2 (7.9)	65.2 (8.6)	62.9 (8.4)	64.7 (7.8)	64.5 (8.0)	64.4 (8.2)
Sex, male	133 (70.0)	129 (72.1)	131 (69.7)	135 (69.9)	132 (70.6)	133 (68.9)	793 (70.2)
Race							
Asian	0	0	0	0	1 (0.5)	0	1 (0.1)
White	190 (100)	179 (100)	188 (100)	193 (100)	186 (99.5)	193 (100)	1129 (99.9)
BMI (kg/m^2^)	26.20 (4.04)	26.36 (3.64)	26.19 (4.03)	26.10 (3.98)	26.04 (3.82)	26.05 (3.73)	26.16 (3.87)
Time since COPD diagnosis (years)	8.9 (6.1)	8.6 (5.9)	9.0 (5.8)	8.3 (5.3)	9.4 (6.4)	8.7 (5.3)	8.8 (5. 8)
Main COPD phenotype^a^							
Chronic bronchitis only	101 (53.2)	93 (52.0)	107 (56.9)	113 (58.5)	106 (56.7)	108 (56.0)	628 (55.6)
Emphysema only	39 (20.5)	41 (22.9)	34 (18.1)	36 (18.7)	36 (19.3)	42 (21.8)	228 (20.2)
Mixed	50 (26.3)	45 (25.1)	47 (25.0)	44 (22.8)	45 (24.1)	43 (22.3)	274 (24.2)
Exacerbations in previous year	1.2 (0.4)	1.1 (0.4)	1.1 (0.3)	1.1 (0.4)	1.1 (0.4)	1.1 (0.4)	1.1 (0.4)
1	160 (84.2)	157 (87.7)	164 (87.2)	173 (89.6)	161 (86.1)	174 (90.2)	989 (87.5)
2	30 (15.8)	19 (10.6)	24 (12.8)	16 (8.3)	24 (12.8)	17 (8.8)	130 (11.5)
> 2	0	3 (1.7)	0	4 (2.1)	2 (1.1)	2 (1.0)	11 (1.0)
Smoking history							
Pack-years	33.4 (15.4)	36.1 (15.1)	36.4 (17.5)	35.2 (15.1)	36.1 (15.0)	36.1 (14.3)	35.5 (15.4)
Ex-smoker	91 (47.9)	85 (47.5)	97 (51.6)	77 (39.9)	82 (43.9)	100 (51.8)	532 (47.1)
Current smoker	99 (52.1)	94 (52.5)	91 (48.4)	116 (60.1)	105 (56.1)	93 (48.2)	598 (52.9)
FEV_1_ (% predicted)^b^	48.7 (10.4)	48.6 (11.2)	47.1 (10.3)	47.9 (10.3)	48.0 (10.6)	48.1 (11.0)	48.1 (10.6)
FEV_1_/FVC ratio^b^	0.454 (0.114)	0.445 (0.109)	0.452 (0.110)	0.445 (0.098)	0.431 (0.100)	0.439 (0.095)	0.444 (0.104)
CAT total score	20.7 (5.1)	20.6 (5.5)	21.0 (4.9)	20.4 (5.0)	20.4 (5.1)	20.2 (5.0)	20.5 (5.1)
mMRC score	2.4 (0.5)	2.4 (0.5)	2.4 (0.5)	2.3 (0.5)	2.3 (0.5)	2.3 (0.5)	2.3 (0.5)

Data are mean (SD) or number (%). ^a^As assessed by the investigator. ^b^Post-bronchodilator. *BID* twice daily, *BMI* body mass index, *COPD* chronic obstructive pulmonary disease, *FEV*_*1*_ forced expiratory volume in 1 s, *FVC* forced vital capacity, *CAT* COPD Assessment Test, *mMRC* modified Medical Research Council dyspnoea scale

### Outcomes

#### Lung function

**Fig. 2:**
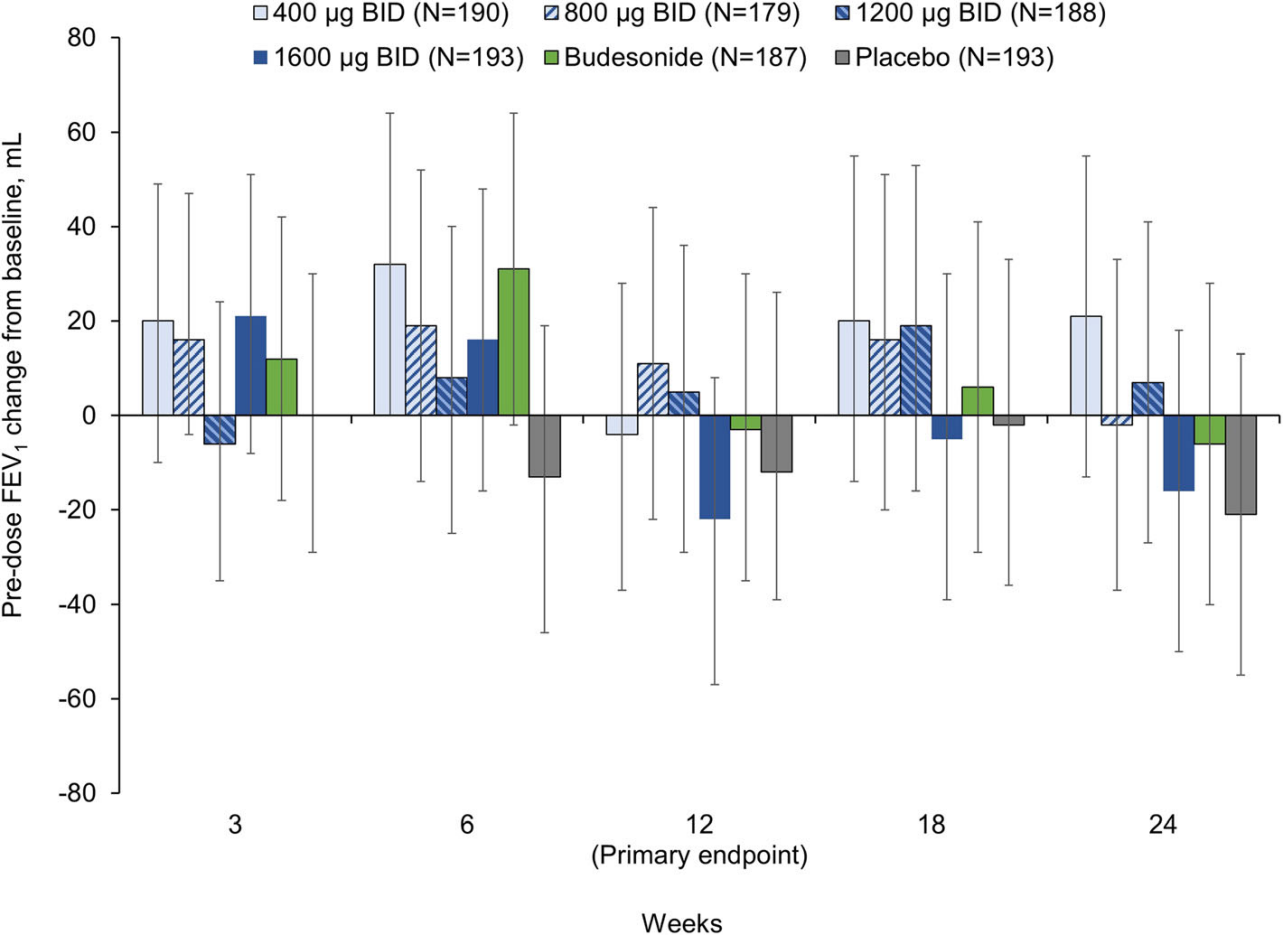
Adjusted mean pre-dose FEV_1_ change from baseline (ITT population). Data are adjusted mean and 95% confidence intervals. FEV_1_, forced expiratory volume in 1 s; ITT, intention-to-treat; BID, twice daily

For the primary endpoint (pre-dose FEV_1_ at Week 12), changes from baseline were small in all groups. There was no clear dose-response for CHF6001 in the linear, E_max_, or linear-log models, with adjusted mean treatment–placebo differences of 2, 17, 10, and −18 mL for CHF6001 400, 800, 1200 and 1600 μg BID, respectively, and 4 mL for budesonide, none of which were statistically significant. Results were similar at other visits, with changes from baseline small, and no significant differences between groups (Fig. 2). Similarly, for pre-dose FVC and IC, changes from baseline were small, and there were no consistent treatment–placebo or CHF6001–budesonide differences, and no CHF6001 dose-response effect (see Additional file 1: Supplementary Figures 1 and 2).

#### Symptoms-related endpoints

There were no consistent treatment–placebo or CHF6001–budesonide differences, with improvements from baseline in the symptoms-related endpoints TDI, SGRQ and E-RS at all timepoints in all groups, including placebo (Table 2, Additional file 1: Supplementary Figures 3–5). Similarly, there were no consistent treatment–placebo or CHF6001–budesonide differences in rescue medication use (see Additional file 1: Supplementary Figures 6 and 7).

**Table 2:** TDI focal score, and SGRQ and E-RS total scores (ITT population)

	CHF6001				Budesonide 800 μg (***N*** = 187)	Placebo (***N*** = 193)
	400 μg BID (***N*** = 190)	800 μg BID (***N*** = 179)	1200 μg BID (***N*** = 188)	1600 μg BID (***N*** = 193)		
**TDI focal score**						
Week 12	1.23 (0.88, 1.57)	1.21 (0.84, 1.57)	1.15 (0.80, 1.50)	0.99 (0.64, 1.34)	1.03 (0.68, 1.37)	1.21 (0.86, 1.56)
Treatment–placebo difference	0.02 (−0.48, 0.51)	−0.00 (−0.51, 0.50)	−0.06 (−0.55, 0.44)	−0.22 (−0.72, 0.28)	−0.18 (−0.68, 0.31)	
Week 24	1.54 (1.19, 1.90)	1.28 (0.90, 1.65)	1.49 (1.13, 1.85)	1.44 (1.08, 1.81)	1.49 (1.13, 1.84)	1.46 (1.11, 1.82)
Treatment–placebo difference	0.08 (−0.42, 0.58)	−0.19 (−0.70, 0.33)	0.03 (−0.48, 0.53)	−0.02 (−0.53, 0.49)	0.02 (−0.48, 0.53)	
**SGRQ total score, change from baseline**						
Week 12	−4.53 (−6.43, −2.62)	−6.62 (−8.57, −4.66)	−4.58 (−6.47, −2.69)	−6.38 (−8.26, −4.50)	−5.56 (−7.45, −3.67)	−6.17 (−8.06, −4.29)
Treatment–placebo difference	1.65 (−1.03, 4.33)	−0.44 (−3.15, 2.27)	1.59 (−1.07, 4.26)	−0.21 (−2.87, 2.46)	0.62 (−2.05, 3.28)	
Week 24	−5.54 (−7.64, −3.43)	−8.06 (−10.24, −5.89)	−5.95 (−8.04, −3.86)	−6.96 (−9.06, −4.87)	−7.11 (−9.20, −5.02)	−7.48 (−9.57, −5.40)
Treatment–placebo difference	1.95 (−1.02, 4.91)	−0.58 (−3.59, 2.43)	1.54 (−1.42, 4.49)	0.52 (−2.43, 3.47)	0.38 (−2.58, 3.33)	
**E-RS total score, change from baseline**						
Overall	−1.53 (−2.12, −0.94)	−2.41 (−3.01, −1.80)	−1.89 (−2.48, −1.31)	−2.07 (−2.66, −1.48)	−2.35 (−2.94, −1.76)	−2.21 (−2.79, −1.63)
Treatment–placebo difference	0.68 (−0.14, 1.50)	−0.20 (−1.03, 0.64)	0.32 (−0.51, 1.14)	0.14 (−0.68, 0.97)	−0.14 (−0.97, 0.68)	

Data are adjusted mean (95% CI). There were no statistically significant treatment–placebo or CHF6001–budesonide differences. *TDI* Transition Dyspnea Index, *SGRQ* St George’s Respiratory Questionnaire, *E-RS* Exacerbations of Chronic Pulmonary Disease Tool – Respiratory Symptoms, *ITT* intention-to-treat, *BID* twice daily

#### Exacerbations

**Fig. 3:**
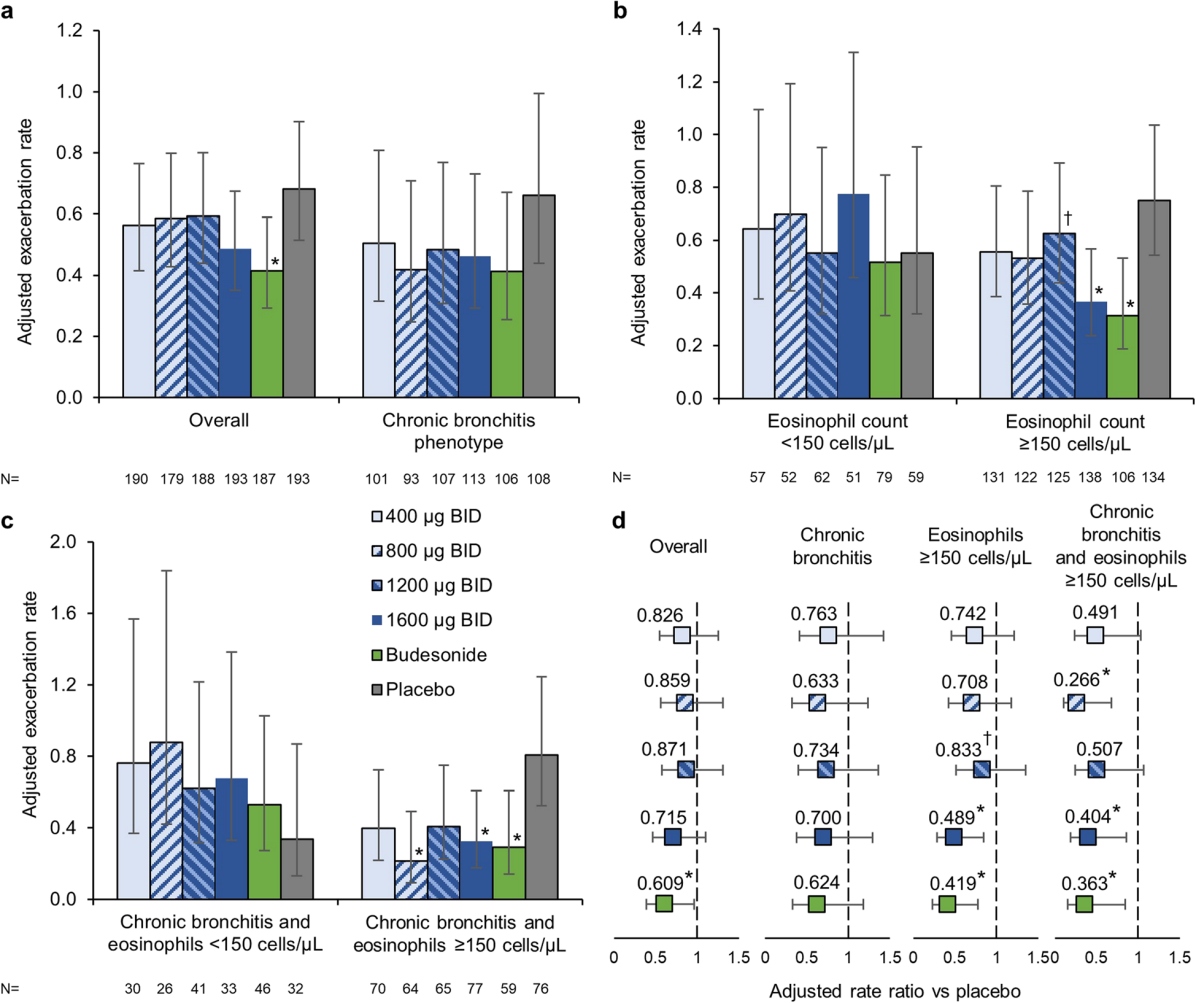
Annualised moderate-to-severe exacerbation rate: **a** In the overall population (pre-specified analysis) and in the subgroup of patients with a chronic bronchitis phenotype (post-hoc analysis); **b** By eosinophil count at baseline (post-hoc analysis); and **c** In the subgroup with a chronic bronchitis phenotype by eosinophil count at baseline (post-hoc analysis). **d** Adjusted rate ratio versus placebo, overall, in the subgroup of patients with a chronic bronchitis phenotype, and in the subgroup of patients with a chronic bronchitis phenotype who also had eosinophil count ≥150 cells/μL at baseline. (All in the ITT population.). Data in Panels **a**, **b** and **c** are adjusted mean and 95% confidence intervals; data in Panel **d** are rate ratios and 95% confidence intervals. *p < 0.05 vs placebo; ^†^*p* < 0.05 vs budesonide. ITT, intention-to-treat; BID, twice daily

The adjusted annualised rates of moderate-to-severe exacerbations were 0.56, 0.59, 0.59 and 0.49 for CHF6001 400, 800, 1200 and 1600 μg BID, respectively, 0.42 for budesonide and 0.68 for placebo (Fig. 3a). Non-statistically significant exacerbation rate reductions were observed with the four CHF6001 treatment groups compared with placebo, ranging from 13 to 28% (Fig. 3d). In the budesonide group there was a 39% reduction in the exacerbation rate compared to placebo (*p* = 0.030).

In the *post-hoc* analysis conducted only in patients with a chronic bronchitis phenotype, the effect of CHF6001 was numerically larger than in the overall analysis, but still statistically non-significant, with rate reductions versus placebo of 24–37% (Fig. 3a and d); the effect of budesonide in this subgroup was numerically similar to that in the overall population (38% reduction versus placebo; non-significant). When analysed according to eosinophil count at baseline, the effect of the four CHF6001 doses was generally larger in patients with blood eosinophil counts ≥150 cells/μL than < 150 cells/μL (especially for 1600 μg BID, where the rate reduction versus placebo was 51% in the ≥150 cells/μL group, *p* = 0.010), as was that of budesonide (58%, *p* = 0.006; Fig. 3b and d). Combining these two subgroups (i.e., patients with a chronic bronchitis phenotype and with eosinophil count ≥150 cells/μL) further increased the treatment effect versus placebo, with rate reductions versus placebo of 49–73% with CHF6001 (reaching statistical significance for the 800 and 1600 μg BID doses, *p* = 0.005 and *p* = 0.019, respectively) and 63% for budesonide (*p* = 0.019; Fig. 3c and d).

#### Biomarkers

**Fig. 4:**
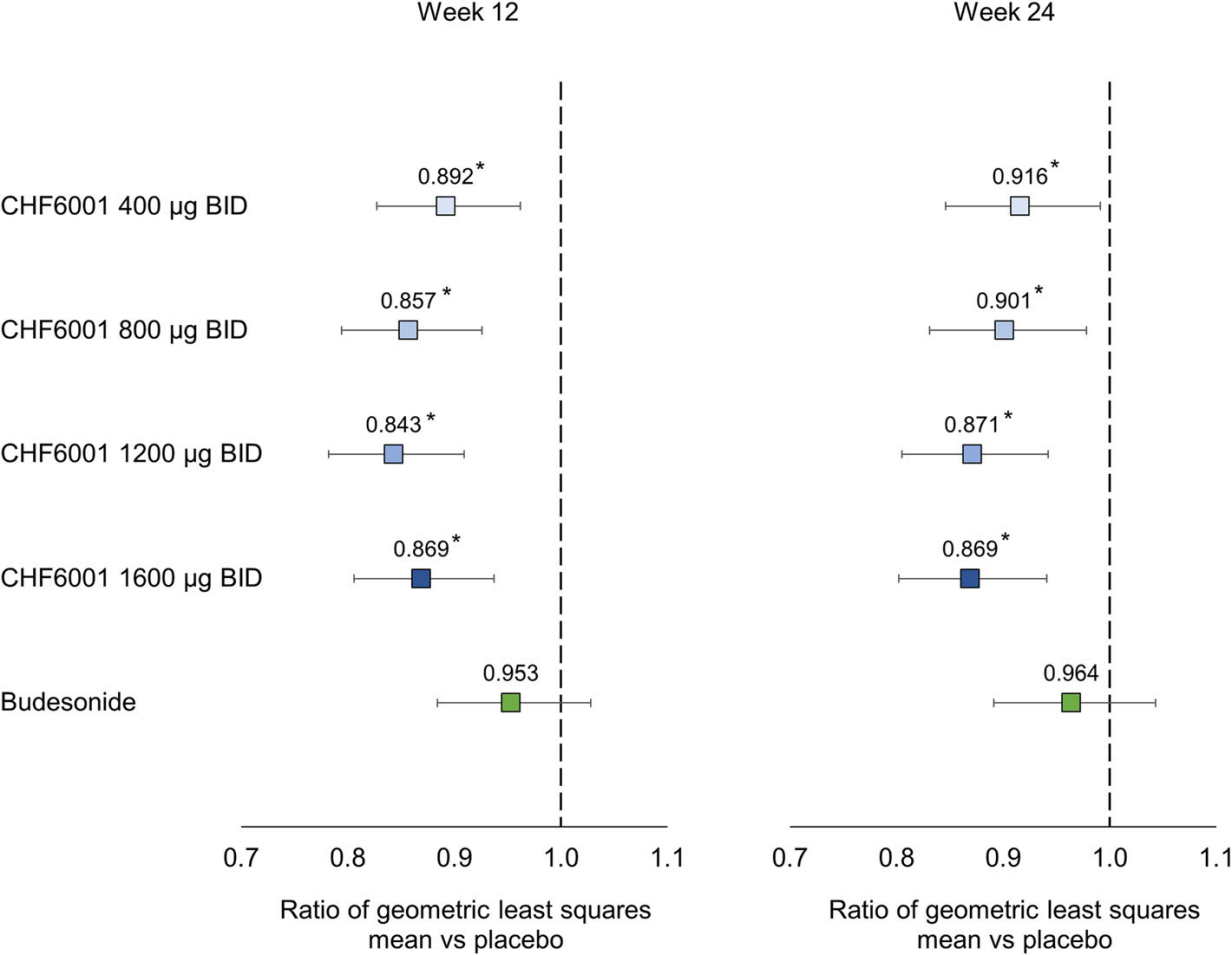
Effect of treatments on surfactant protein D (SP-D) – geometric least squares mean ratio versus placebo (ITT population). *p < 0.05 vs placebo. ITT, intention-to-treat; BID, twice daily

All four CHF6001 doses significantly reduced the levels of SP-D versus placebo (with no indication of dose-response), whereas the levels were unchanged in the budesonide group (Fig. 4). None of the treatments had consistent effects on any of the other blood biomarkers with the exception of blood eosinophil levels, where budesonide significantly decreased counts versus placebo at both Week 12 and Week 24, whereas CHF6001 did not (see Additional file 1: Supplementary Table 1).

### Safety

All treatments were similarly well tolerated, with no CHF6001 dose-effect, few AEs considered related to treatment, and most AEs being mild or moderate in severity (Table 3). The only severe or serious AE to occur in more than one patient in any group was COPD exacerbation (Additional file 1: Supplementary Table 2). No severe or serious AEs were considered related to treatment. Eight patients withdrew due to AEs that were considered treatment related (one or two patients in each active treatment group and three with placebo) – the AEs that led to withdrawal from the CHF6001 groups were all mild or moderate in severity. None of the deaths that occurred during the study were considered treatment related: CHF6001 400 μg, one patient with two AEs (COPD exacerbation and pneumonia); 800 μg: one patient (AE reported only as ‘death’); 1200 μg: one patient (cardiac arrest); 1600 μg: two patients, one with two AEs (coronary artery disease and COPD exacerbation), and one with AE reported only as ‘death’. The incidence of pneumonia AEs was low and similar in all groups, with no treatment-related events. The incidence of AEs of special interest (which included diarrhoea and weight loss) was low, and similar in all groups (see Additional file 1: Supplementary Table 2).

**Table 3:** Overall adverse events and drug-related adverse events (safety population)

	CHF6001				Budesonide 800 μg (***N*** = 187)	Placebo (***N*** = 193)
	400 μg BID (***N*** = 190)	800 μg BID (***N =*** 179)	1200 μg BID (***N*** = 188)	1600 μg BID (***N*** = 193)		
Adverse events	91 (47.9)	96 (53.6)	94 (50.0)	84 (43.5)	94 (50.3)	103 (53.4)
Drug-related adverse events	10 (5.3)	3 (1.7)	6 (3.2)	6 (3.1)	8 (4.3)	14 (7.3)
Severe adverse events	8 (4.2)	10 (5.6)	7 (3.7)	4 (2.1)	6 (3.2)	5 (2.6)
Serious adverse events	11 (5.8)	13 (7.3)	12 (6.4)	7 (3.6)	10 (5.3)	7 (3.6)
Serious drug-related adverse events	0	0	0	0	0	0
Adverse events leading to study drug discontinuation	5 (2.6)	3 (1.7)	4 (2.1)	8 (4.1)	2 (1.1)	5 (2.6)
Adverse events leading to death	1 (0.5)	1 (0.6)	1 (0.5)	2 (1.0)	0	0

*BID* twice daily

There were no treatment-related trends in biochemistry, haematology, urinalysis or blood pressure data. Mean changes from baseline in heart rate were small and clinically insignificant, as were changes in QTcF interval, with no trends in QTcF interval notable values or notable changes. There were no significant mean changes in bodyweight, and no treatment-related changes in bodyweight or appetite.

## Discussion

CHF6001 had no effect on the primary endpoint measurement of FEV_1_ at 12 weeks. All four CHF6001 doses demonstrated a similar good overall safety and tolerability profile. CHF6001 did not have a significant effect on moderate-to-severe exacerbations in the overall population. However, the relative effect of CHF6001 versus placebo on exacerbations was numerically larger in the hypothesis-generating *post-hoc* analysis of patients with a chronic bronchitis phenotype than in the overall population (although still not significant), and was larger still in the subgroup with both a chronic bronchitis phenotype and baseline blood eosinophil count ≥150 cells/μL, reaching statistical significance for the 800 and 1600 μg BID doses.

The good tolerability profile of CHF6001 (including for gastrointestinal adverse events) is notable since treatment with roflumilast, the oral PDE4 inhibitor, is associated with marked gastrointestinal adverse events that has been reported to materially impact patient tolerability [1, 3–7, 13]. Indeed, CHF6001 has been specifically designed to minimise systemic exposure, with inhaled dosing clearly more directly targeting the lungs. Although five patients receiving CHF6001 had AEs that resulted in death, none of these AEs was considered related to treatment by the investigators.

The greater effect of CHF6001 in a defined COPD subgroup is similar to that observed with roflumilast. In an initial one-year study conducted in a broad COPD population, roflumilast improved lung function versus placebo by 39 mL at 52 weeks (*p* = 0.001) but there was no effect on exacerbations [14]. However, in a subsequent *post-hoc* analysis pooling data from this study and a second 12-month study, the greatest effect of roflumilast on exacerbations was in the subset of patients with chronic bronchitis (with or without emphysema), in whom there was a 26% rate reduction versus placebo (*p* = 0.001) [15]. This finding led to the execution of studies that specifically recruited patients with chronic bronchitis, in which roflumilast reduced the exacerbation rate versus placebo by 17% (*p* = 0.0003) [13]. Furthermore, in patients with chronic bronchitis, the benefit of roflumilast increased with increasing baseline blood eosinophil count [16], and administration of roflumilast significantly reduced eosinophil cell counts in bronchial biopsy samples and induced sputum, suggesting that the efficacy of roflumilast could be due at least in part to an impact on lung eosinophils [17].

The lack of effect in the current study on the lung function endpoints of any of the active treatments (including the ICS positive control) was surprising. This was perhaps partly due to the high variability in these endpoints (as indicated by the wide confidence intervals), with greater variability than seen previously with other PDE4 inhibitors [18, 19]. In addition, there were no consistent treatment–placebo or CHF6001–budesonide differences in any of the symptoms-related endpoints, although there were marked improvements from baseline for these endpoints in all groups including placebo, with mean changes from baseline being close to, or exceeding clinical relevance for TDI (1 unit), SGRQ (4 units) and E-RS (2 units) at later timepoints. It is important to note that the placebo group received formoterol, which was also administered during the run-in period, and so minimal or no changes were expected during the treatment period in this group. Furthermore, all patients were receiving a LABA plus an ICS before the study, with the ICS withdrawn at the start of the run-in period. Overall, this suggests that a ‘trial effect’ influenced these endpoints, as such improvements in symptoms with placebo could not be due to treatment itself. A number of studies of other PDE4 inhibitors in patients with COPD have also shown improvements from baseline in symptoms in the placebo arm (although generally modest), but were still able to show differences between active and placebo treatments [3, 13]. Previous studies have also shown a benefit of budesonide plus formoterol compared with formoterol alone on symptoms [20–23]. These contrasting results make the anti-inflammatory effect of CHF6001 and budesonide on these endpoints challenging to interpret.

In terms of the biomarkers, SP-D levels were decreased with all four CHF6001 doses but not budesonide, suggesting that this is a PDE4 inhibitor effect and not an ICS effect. SP-D is a secretory product of non-ciliated bronchiolar cells [24], circulating levels of which are a biomarker of lung injury, suggesting an active involvement in surfactant metabolism and/or host defence within small airways. This is particularly important in view of the extrafine formulation of CHF6001, which might have the potential to decrease SP-D leakage from the small airways to the systemic circulation and improve small airways integrity. Furthermore, in patients with COPD decreases in circulating SP-D are associated with improvements in health status [25, 26]. A reduction in SP-D levels was also observed in a previous study in which CHF6001 was administered on top of inhaled triple therapy in patients with a chronic bronchitis phenotype [10]. It is possible that the impact of CHF6001 on SP-D indicates a relevant pharmacological effect that is associated with prevention of COPD deterioration, although this needs to be confirmed in larger, longer studies.

The main limitation was that this study was designed (and powered) to support selection of the optimal CHF6001 dose in terms of effect on lung function, including a 24-week treatment duration. The primary objective was not achieved, and so care needs to be taken over the interpretation of the other data. In addition, the most interesting data are from *post-hoc* analyses of moderate-to-severe exacerbations, and so by their nature are exploratory, being unpowered and with no correction for multiplicity; a suitably designed prospective study is needed to confirm these data. Of note, even though eligible patients were required to have a history of at least one exacerbation in the previous 12 months, the rates of these events during the follow-up period was relatively low.

## Conclusions

In conclusion, in this study CHF6001 was well tolerated with a good overall safety profile, but had no effect in the primary lung function analysis and the optimal dose was not identified. However, the *post-hoc* analyses indicate specific subgroups of patients who may receive particular benefit from CHF6001. Future studies of PDE4 inhibitors should be targeted at these subgroups.

## Supplementary information

**Additional file 1.** Supplementary methods and results. Methods and results supporting main body of the manuscript.

## Data Availability

Chiesi commits to sharing with qualified scientific and medical Researchers, conducting legitimate research, patient-level data**,** study-level data, the clinical protocol and the full clinical study report of Chiesi Farmaceutici S.p.A.-sponsored interventional clinical trials in patients for medicines and indications approved by the European Medicines Agency and/or the US Food and Drug Administration after *1st January 2015*, following the approval of any received research proposal and the signature of a Data Sharing Agreement. Chiesi provides access to clinical trial information consistently with the principle of safeguarding commercially confidential information and patient privacy. To date, the current study is out of scope of the Chiesi policy on Clinical Data Sharing. Other information on Chiesi’s data sharing commitment, access and research request’s approval process are available in the Clinical Trial Transparency section of http://www.chiesi.com/en/research-and-development/.
