# Supplementary material for: Efficacy and safety of CHF6001, a novel inhaled PDE4 inhibitor in COPD: the PIONEER study
Source: Respir Res. 2020 Sep 22;21:246. doi: 10.1186/s12931-020-01512-y (PMC7510119; doi:10.1186/s12931-020-01512-y)
Supplement: Supplementary file 1 — Additional file 1. Supplementary methods and results. Methods and results supporting main body of the manuscript. [file 12931_2020_1512_MOESM1_ESM.docx]

# Efficacy and safety of CHF6001, a novel inhaled PDE4 inhibitor in COPD: the PIONEER study

Dave Singh, Aida Emirova, Catherine Francisco, Debora Santoro, Mirco Govoni, Marie Anna Nandeuil

# Additional file 1

## Methods

### Inclusion criteria

For inclusion into the study, patients were required to fulfil all of the following criteria:

1. Male and female aged ≥ 40 years with written informed consent obtained prior to any study-related procedure;
2. A female was eligible to enter the study if she was of non-child bearing potential (i.e. physiologically incapable of becoming pregnant) (e.g. postmenopausal women defined as being amenorrhoeic for ≥ 12 consecutive months without an alternative medical cause) or women permanently sterilised (e.g. bilateral oophorectomy, hysterectomy or bilateral salpingectomy).

Women physiologically capable of becoming pregnant (i.e. women of child bearing potential) were eligible to enter the study if non-breast-feeding women and if they had a negative pregnancy test at screening and agreed to use one or more of the following highly effective contraceptive measures:

- Placement of an intrauterine device or intrauterine hormone-releasing system;
- Combined (oestrogen and progestogen containing) hormonal contraception associated with inhibition of ovulation (oral, intravaginal, transdermal);
- Progesterone-only hormonal contraception associated with inhibition of ovulation (oral, injectable, implantable);
- Bilateral tubal occlusion;
- Vasectomised partner.

Reliable contraception was to be maintained throughout the study.

Abstinence was acceptable where it was in line with the patient’s preferred and usual lifestyle.

Pregnancy tests were performed at study entry (a serum test at the screening visit and a urine test at screening and randomisation visits) in all women of child bearing potential;

1. With an established diagnosis of COPD (according to Global Initiative for Chronic Obstructive Lung Disease [GOLD] guidelines, 2015 update) at least 12 months prior to the screening visit;
2. With a smoking history of at least 10 pack‑years (pack-years = [number of cigarettes per day x number of years]/20). Current and ex‑smokers were eligible.
   Smoking cessation should have been at least 3 months prior to the screening visit. If the patients underwent smoking cessation therapy, it should have been completed 3 months prior to the screening visit;
3. With a post-bronchodilator forced expiratory volume in 1 second (FEV_1_) ≥30% and ≤70% of the patient predicted normal value and a post‑bronchodilator FEV_1_/forced vital capacity (FVC) ratio < 0.7 measured 10‑15 minutes after administration of 400 µg (4 puffs x 100 µg) of salbutamol pressured metered dose inhaler (pMDI). If this criterion was not met at screening, the test could be repeated once before the randomisation visit;
4. With a documented history (e.g. medical record verification) of at least one moderate or severe exacerbation in the 12 months prior to study entry. Patient verbal reports were not acceptable.

COPD exacerbation was defined according to the following:

“A sustained worsening of the patient’s condition (dyspnoea, cough and/or sputum production/purulence), from the stable state and beyond normal day-to-day variations, that was acute in onset and necessitated a change in regular medication in a patient with underlying COPD that included prescriptions of systemic corticosteroids (oral/intravenous [IV]/intramuscular [IM] corticosteroids) and/or antibiotics (for a moderate exacerbation) or need for hospitalisation (for severe exacerbation)”.

Also documented visits to an emergency department due to COPD exacerbation were considered acceptable to fulfil this criterion;

1. Patients should have been symptomatic at screening (defined as having a modified Medical Research Council dyspnoea [mMRC] score ≥ 2 and a COPD Assessment Test [CAT] score ≥ 10);
2. Patients should have been receiving daily maintenance therapy with an inhaled corticosteroid (ICS) and a long-acting β_2_-agonist (LABA) only, at stable dose and dosage regimen, for at least 2 months prior to screening;
3. Patients should have had a cooperative attitude and ability to be trained to use correctly the dry powder inhalers (DPI; NEXThaler^®^, Turbohaler^®^);
4. Patients should have had a cooperative attitude and ability to be trained to use correctly the electronic devices with COPD questionnaires, to understand and to perform required outcome measurements of the protocol (e.g. spirometry manoeuvres) and ability to understand the risks involved.

Inclusion criterion 2 was verified again at the randomisation visit (V2, Week 0).

### Exclusion Criteria

The presence of any of the following excluded a patient from study enrolment:

1. Patients with a diagnosis of asthma or other respiratory disorders (other than COPD) which may have interfered with data interpretation according to the Investigator’s opinion;
2. Patients on maintenance bronchodilators therapy only (LABA alone, long-acting muscarinic antagonist [LAMA] alone, dual LABA/LAMA) within 8 weeks prior to screening;
3. Patients on maintenance triple therapy (ICS/LABA plus LAMA) or LAMA plus ICS combination therapy, or on phosphodiesterase 4 (PDE4) inhibitors (e.g. roflumilast) within 8 weeks prior to screening;
4. Patients with a moderate or severe COPD exacerbation (i.e. resulting in the use of systemic corticosteroids [oral/IV/IM corticosteroids] and/or antibiotics or need for hospitalisation) or a lower respiratory tract infection within 6 weeks prior to study entry or during the run-in period;
5. Patients requiring long term (at least 12 hours daily) oxygen therapy for chronic hypoxemia;
6. Patients who were participating to a pulmonary rehabilitation programme or completing such a programme within the last 6 weeks prior to screening;
7. Patients with known respiratory disorders other than COPD that in the Investigator’s opinion, would have affected efficacy and safety evaluation or place the patient at risk. This included but was not limited to known α‑1 antitrypsin deficiency, active tuberculosis, bronchiectasis, sarcoidosis, lung fibrosis, pulmonary hypertension and interstitial lung disease;
8. Patients with lung cancer or a history of lung cancer;
9. Patients with active cancer or a history of cancer with less than 5 years disease‑free survival time (whether or not there was evidence of local recurrence or metastases). Localised carcinoma (e.g. basal cell carcinoma without metastases, in situ carcinoma of the cervix adequately treated) was acceptable;
10. Patients with a history of hypersensitivity to β_2_‑agonist, corticosteroids, PDE4 inhibitors or any of the excipients contained in any of the formulations used in the study;
11. Patients with a diagnosis of depression, generalised anxiety disorder, suicidal ideation or behaviour that would have, according to the Investigator judgement, placed the patients at undue risk;
12. Patients who had clinically significant (CS) cardiovascular condition such as, but not limited to, unstable ischemic heart disease, New York Heart Association Class III/IV heart failure, acute ischemic heart disease within one year prior to study entry, known history of atrial fibrillation or a history of sustained and non-sustained cardiac arrhythmias diagnosed in the last 6 months not controlled with a rate control strategy;
13. Patients having a clinically abnormal significant 12‑lead electrocardiogram (ECG) that, in the Investigator’s opinion, would have affected efficacy or safety evaluation or placed the patient at risk.

Male patients with a QT interval corrected using Fridericia’s formula (QTcF)>450 msec and female patients with a QTcF >470 msec at screening and/or at randomisation visits were not eligible;

1. Patients with a serum potassium value ≤3.5 mEq/L or >5.5 mEq/L and/or a fasting serum glucose value ≥ 140 mg/dL. In case of non‑interpretable data, another determination was performed as soon as possible and prior to randomisation (V2, Week 0);
2. Patients with a history or symptoms of significant neurological disease including transient ischemic attack, stroke, seizure disorder or behavioural disturbances;
3. Patients who had unstable concurrent disease: e.g. uncontrolled thyroid disease, uncontrolled diabetes mellitus or other endocrine disease; significant renal impairment; history of cerebrovascular disease uncontrolled gastrointestinal disease (e.g. active peptic ulcer, Crohn’s disease, ulcerative colitis, enteritis, unexplained diarrhoea, bloody or loose stools); uncontrolled haematological disease; uncontrolled autoimmune disorders (e.g. rheumatoid arthritis, inflammatory bowel disease**)**, or other disease or other condition that could have, in the judgement of the Investigator, placed the patient at undue risk or potentially compromised the results or interpretation of the study;
4. Patients with CS laboratory abnormalities indicating a significant or unstable concomitant disease that would have, in the judgement of the Investigator, placed the patient at undue risk or potentially compromised the results or interpretation of the study;
5. Patients with abnormal alanine aminotransferase (ALT) ≥ 2 x upper limit of normal (ULN) and/or aspartate aminotransferase (AST) ≥ 2 x ULN and/or bilirubin ≥ 1.5 x ULN. Isolated bilirubin ≥ 1.5 x ULN was acceptable if fractionated and direct bilirubin was < 35%;
6. Current or chronic history of liver disease or known hepatic or biliary abnormalities (with the exception of Gilbert’s syndrome or asymptomatic gallstones).
7. Patients who were receiving treatment with any drug known to have a well‑defined potential for hepatotoxicity (e.g. isoniazide, nimesulide, ketoconazole) within the previous 3 months before the screening visit;
8. Patients who were severely obese (body mass index [BMI] ≥ 35 kg/m^2^) or who had experienced excessive weight loss recently (which could not be explained by the natural course of COPD or known background conditions);
9. Patients with a history of alcohol abuse and/or substance/drug abuse within 12 months prior to screening visit;
10. Patients having received any other investigational drug within the preceding 30 days (60 days for biologics), or a longer and more appropriate time as determined by the Investigator (e.g. approximately five half-lives of the previous investigational drug).

Exclusion criteria 4 and 13 were re-checked at the randomisation visit (V2, Week 0).

## Results

Supplementary Figure 1. Adjusted mean pre-dose FVC change from baseline (ITT population).


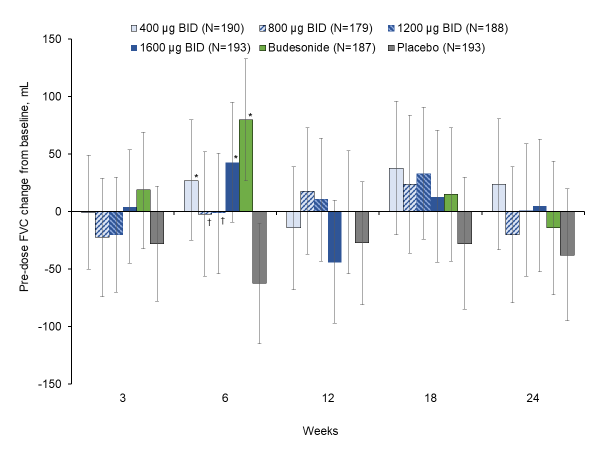


Data are adjusted mean and 95% confidence intervals. *p<0.05 vs placebo; ^†^p<0.05 vs budesonide. FVC, forced vital capacity; ITT, intention-to-treat; BID, twice daily.

Supplementary Figure 2. Adjusted mean pre-dose IC change from baseline (ITT population).


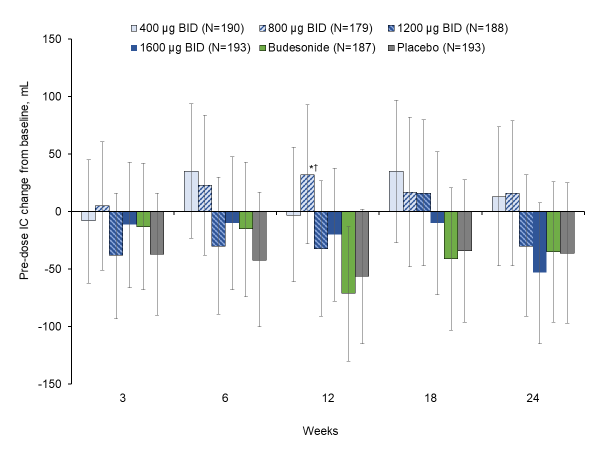


Data are adjusted mean and 95% confidence intervals. *p<0.05 vs placebo; ^†^p<0.05 vs budesonide. IC, inspiratory capacity; ITT, intention-to-treat; BID, twice daily.

Supplementary Figure 3. Adjusted mean TDI focal score (ITT population).


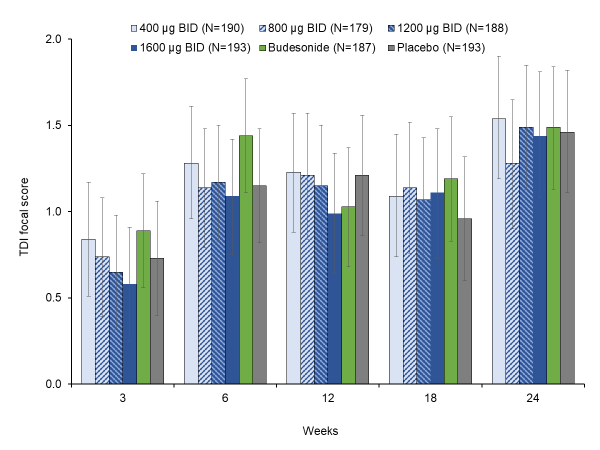


Data are adjusted mean and 95% confidence intervals. TDI, Transition Dyspnea Index; ITT, intention-to-treat; BID, twice daily.

Supplementary Figure 4. Adjusted mean SGRQ total score change from baseline (ITT population).


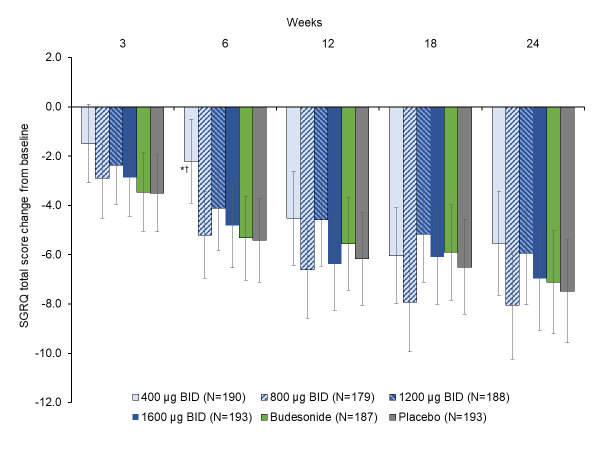


Data are adjusted mean and 95% confidence intervals. *p<0.05 vs placebo; ^†^p<0.05 vs budesonide. SGRQ, St George’s Respiratory Questionnaire; ITT, intention-to-treat; BID, twice daily.

Supplementary Figure 5. Adjusted mean E-RS total score change from baseline (ITT population).


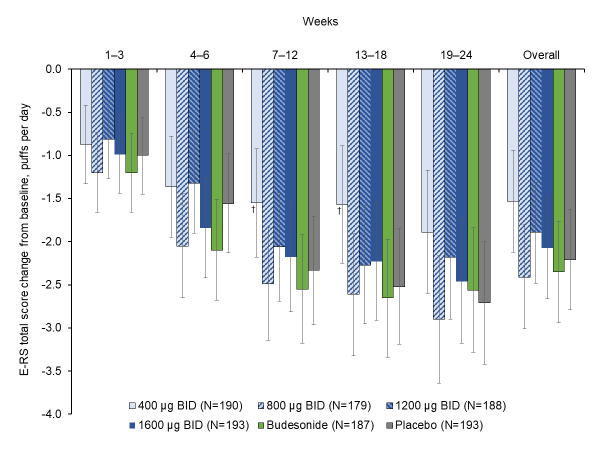


Data are adjusted mean and 95% confidence intervals. E-RS, Exacerbations of Chronic Pulmonary Disease Tool – Respiratory Symptoms; ITT, intention-to-treat; BID, twice daily.

Supplementary Figure 6. Adjusted mean percentage of days without rescue medication, change from baseline (ITT population).


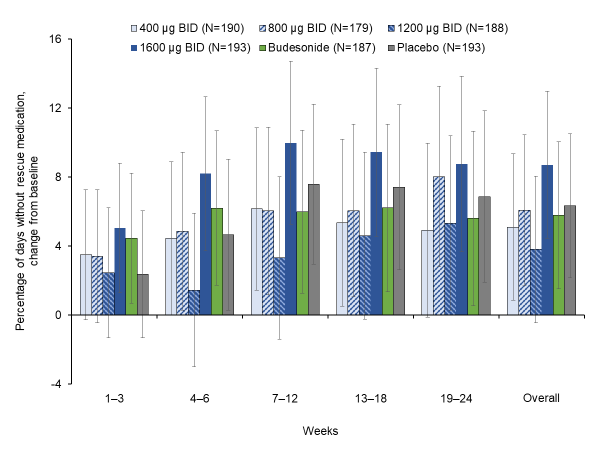


Data are adjusted mean and 95% confidence intervals. ITT, intention-to-treat; BID, twice daily.

Supplementary Figure 7. Adjusted mean rescue medication use change from baseline (ITT population).


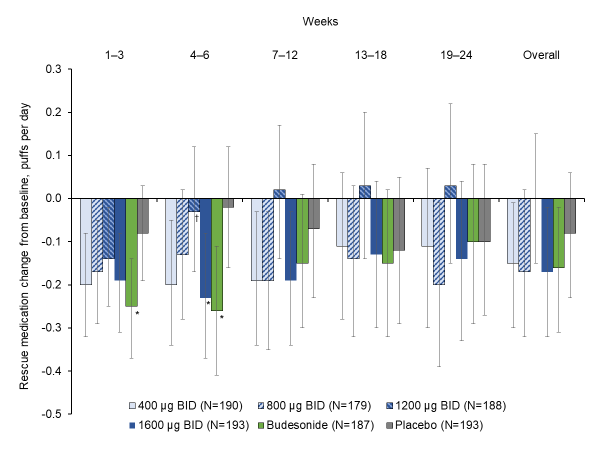


Data are adjusted mean and 95% confidence intervals. *p<0.05 vs placebo; ^†^p<0.05 vs budesonide. ITT, intention-to-treat; BID, twice daily

Supplementary Table 1. Effect of treatments on blood eosinophil count, and blood biomarkers (ITT population).

|  | **CHF6001** | | | | **Budesonide 800 µg (N=187)** | **Placebo (N=193)** |
| --- | --- | --- | --- | --- | --- | --- |
|  | **400 µg BID (N=190)** | **800 µg BID (N=179)** | **1200 µg BID (N=188)** | **1600 µg BID (N=193)** |  |  |
| **C-reactive protein (mg/L)** | | |  |  |  |  |
| Week 12 | 0.926 (0.799, 1.074) | 1.113 (0.954, 1.300) | 1.162 (1.003, 1.347) | 1.115 (0.962, 1.292) | 0.856 (0.739, 0.991) | 0.965 (0.832, 1.119) |
| Ratio vs placebo | 0.960 (0.778, 1.183) | 1.154 (0.931, 1.430) | 1.205 (0.977, 1.485) | 1.155 (0.937, 1.424) | 0.887 (0.720, 1.093) |  |
| Week 24 | 0.844 (0.715, 0.996) | 1.021 (0.858, 1.214) | 1.248 (1.059, 1.471) | 1.157 (0.979, 1.368) | 0.942 (0.799, 1.110) | 0.984 (0.834, 1.160) |
| Ratio vs placebo | 0.858 (0.679, 1.084) | 1.038 (0.817, 1.318) | 1.269 (1.005, 1.601)* | 1.176 (0.930, 1.488) | 0.958 (0.759, 1.209) |  |
| **Fibrinogen (mg/dL)** |  |  |  |  |  |  |
| Week 12 | 0.909 (0.772, 1.069) | 0.967 (0.815, 1.148) | 0.978 (0.831, 1.150) | 0.842 (0.716, 0.990) | 0.930 (0.791, 1.092) | 1.006 (0.855, 1.184) |
| Ratio vs placebo | 0.903 (0.718, 1.137) | 0.961 (0.759, 1.218) | 0.972 (0.772, 1.223) | 0.837 (0.665, 1.053) | 0.924 (0.735, 1.162) |  |
| Week 24 | 0.885 (0.730, 1.074) | 0.922 (0.753, 1.129) | 0.976 (0.806, 1.182) | 1.002 (0.825, 1.218) | 0.845 (0.698, 1.024) | 0.959 (0.792, 1.162) |
| Ratio vs placebo | 0.923 (0.703, 1.211) | 0.961 (0.727, 1.271) | 1.018 (0.776, 1.334) | 1.045 (0.795, 1.373) | 0.881 (0.672, 1.155) |  |
| **Club cell protein 16** |  |  |  |  |  |  |
| Week 12 | 1.032 (0.984, 1.082) | 1.089 (1.036, 1.144) | 1.040 (0.992, 1.090) | 1.044 (0.996, 1.095) | 1.049 (1.001, 1.099) | 1.063 (1.014, 1.114) |
| Ratio vs placebo | 0.971 (0.908, 1.038) | 1.025 (0.957, 1.098) | 0.978 (0.915, 1.046) | 0.983 (0.919, 1.051) | 0.987 (0.923, 1.055) |  |
| Week 24 | 1.219 (1.147, 1.296) | 1.291 (1.212, 1.376) | 1.241 (1.168, 1.318) | 1.167 (1.098, 1.241) | 1.221 (1.149, 1.297) | 1.215 (1.144, 1.291) |
| Ratio vs placebo | 1.004 (0.921, 1.094) | 1.063 (0.973, 1.160) | 1.021 (0.938, 1.112) | 0.961 (0.881, 1.047) | 1.005 (0.923, 1.095) |  |
| **Interleukin 6 (pg/mL)** |  |  |  |  |  |  |
| Week 12 | 0.932 (0.805, 1.078) | 0.986 (0.845, 1.150) | 1.006 (0.871, 1.161) | 1.092 (0.947, 1.259) | 0.804 (0.698, 0.926) | 0.994 (0.860, 1.148) |
| Ratio vs placebo | 0.938 (0.763, 1.152) | 0.992 (0.803, 1.226) | 1.012 (0.826, 1.241) | 1.099 (0.897, 1.347) | 0.809 (0.661, 0.991)* |  |
| Week 24 | 1.058 (0.915, 1.222) | 1.243 (1.068, 1.446) | 1.405 (1.221, 1.617) | 1.232 (1.067, 1.422) | 1.065 (0.925, 1.227) | 1.155 (1.002, 1.332) |
| Ratio vs placebo | 0.915 (0.747, 1.121) | 1.076 (0.874, 1.325) | 1.216 (0.996, 1.485) | 1.066 (0.871, 1.305) | 0.922 (0.754, 1.127) |  |
| **Interleukin 8 (pg/mL)** |  |  |  |  |  |  |
| Week 12 | 0.959 (0.874, 1.052) | 0.920 (0.834, 1.014) | 0.995 (0.907, 1.091) | 0.995 (0.907, 1.091) | 0.943 (0.860, 1.034) | 1.012 (0.922, 1.110) |
| Ratio vs placebo | 0.948 (0.831, 1.081) | 0.909 (0.795, 1.040) | 0.983 (0.862, 1.121) | 0.983 (0.863, 1.121) | 0.932 (0.818, 1.063) |  |
| Week 24 | 0.946 (0.872, 1.027) | 0.931 (0.855, 1.014) | 0.982 (0.906, 1.064) | 1.050 (0.967, 1.140) | 0.966 (0.891, 1.048) | 0.994 (0.916, 1.077) |
| Ratio vs placebo | 0.952 (0.849, 1.068) | 0.937 (0.833, 1.054) | 0.988 (0.881, 1.108) | 1.057 (0.941, 1.186) | 0.972 (0.867, 1.090) |  |
| **Blood eosinophil count (10^9^/L)** |  |  |  |  |  |  |
| Week 12 | 1.078 (0.972, 1.196) | 0.970 (0.870, 1.081) | 1.012 (0.912, 1.121) | 0.978 (0.883, 1.083) | 0.896 (0.809, 0.993) | 1.087 (0.981, 1.204) |
| Ratio vs placebo | 0.992 (0.858, 1.148) | 0.892  (0.769, 1.036) | 0.931 (0.805, 1.076) | 0.899 (0.778, 1.039) | 0.825 (0.714, 0.953)* |  |
| Week 24 | 1.089 (0.979, 1.212) | 0.997 (0.892, 1.115) | 1.037 (0.933, 1.152) | 0.986 (0.885, 1.097) | 0.886 (0.796, 0.985) | 1.030 (0.926, 1.145) |
| Ratio vs placebo | 1.058 (0.910, 1.230) | 0.968 (0.830, 1.130) | 1.007 (0.867, 1.169) | 0.957 (0.823, 1.113) | 0.860 (0.740, 0.999)* |  |

Data are geometric least squares means (95% CI). *p<0.05 vs placebo. ITT, intention-to-treat; BID, twice daily

Supplementary Table 2. Most common adverse events and drug-related adverse events (safety population).

|  | **CHF6001** | | | | **Budesonide 800 µg (N=187)** | **Placebo (N=193)** |
| --- | --- | --- | --- | --- | --- | --- |
|  | **400 µg BID (N=190)** | **800 µg BID (N=179)** | **1200 µg BID (N=188)** | **1600 µg BID (N=193)** |  |  |
| Adverse events |  |  |  |  |  |  |
| COPD exacerbation | 45 (23.7) | 46 (25.7) | 58 (30.9) | 43 (22.3) | 44 (23.5) | 62 (32.1) |
| Headache | 10 (5.3) | 7 (3.9) | 7 (3.7) | 11 (5.7) | 10 (5.3) | 8 (4.1) |
| Nasopharyngitis | 7 (3.7) | 14 (7.8) | 11 (5.9) | 12 (6.2) | 9 (4.8) | 13 (6.7) |
| Back pain | 5 (2.6) | 3 (1.7) | 5 (2.7) | 4 (2.1) | 3 (1.6) | 2 (1.0) |
| Hyperglycaemia | 5 (2.6) | 2 (1.1) | 2 (1.1) | 1 (0.5) | 1 (0.5) | 1 (0.5) |
| Blood pressure increased | 1 (0.5) | 4 (2.2) | 2 (1.1) | 4 (2.1) | 6 (3.2) | 6 (3.1) |
| AAT increased | 0 | 1 (0.6) | 5 (2.7) | 2 (1.0) | 0 | 2 (1.0) |
| Drug-related adverse events | |  |  |  |  |  |
| Cough | 1 (0.5) | 0 | 1 (0.5) | 2 (1.0) | 0 | 1 (0.5) |
| Dyspnoea | 0 | 0 | 0 | 1 (0.5) | 0 | 2 (1.0) |
| Nausea | 1 (0.5) | 1 (0.6) | 2 (1.1) | 1 (0.5) | 1 (0.5) | 0 |
| Decreased appetite | 2 (1.1) | 0 | 1 (0.5) | 0 | 0 | 0 |
| Severe adverse events | |  |  |  |  |  |
| COPD exacerbation | 4 (2.1) | 5 (2.8) | 5 (2.7) | 2 (1.0) | 2 (1.1) | 4 (2.1) |
| Serious adverse events | |  |  |  |  |  |
| COPD exacerbation | 6 (3.2) | 7 (3.9) | 7 (3.7) | 2 (1.0) | 5 (2.7) | 5 (2.6) |
| Adverse events leading to discontinuation of study drug | | | | |  |  |
| COPD exacerbation | 2 (1.1) | 1 (0.6) | 2 (1.1) | 1 (0.5) | 1 (0.5) | 1 (0.5) |
| Dyspnoea | 0 | 0 | 0 | 2 (1.0) | 0 | 1 (0.5) |
| Adverse events of special interest | | |  |  |  |  |
| Nausea | 1 (0.5) | 2 (1.1) | 2 (1.1) | 2 (1.0) | 1 (0.5) | 0 |
| Abdominal pain upper | 2 (1.1) | 0 | 2 (1.1) | 1 (0.5) | 1 (0.5) | 0 |
| Dyspepsia | 1 (0.5) | 0 | 0 | 2 (1.0) | 1 (0.5) | 1 (0.5) |
| Dry mouth | 2 (1.1) | 0 | 1 (0.5) | 0 | 0 | 1 (0.5) |
| Toothache | 2 (1.1) | 0 | 1 (0.5) | 0 | 0 | 0 |
| Vomiting | 0 | 1 (0.6) | 2 (1.1) | 0 | 0 | 0 |
| Decreased appetite | 2 (1.1) | 0 | 1 (0.5) | 1 (0.5) | 0 | 0 |
| Insomnia | 1 (0.5) | 2 (1.1) | 2 (1.1) | 1 (0.5) | 1 (0.5) | 2 (1.0) |

The most common adverse events are preferred terms occurring in ≥2.5% of patients in any group; the most common drug-related, severe, serious, adverse events leading to discontinuation of study drug, and adverse events of special interest are preferred terms occurring in more than one patient in any group. BID, twice daily; AAT, Aspartate aminotransferase.
